# Spatiotemporal clustering and epidemiological characterization of suicides in Ecuador, 2015–2023

**DOI:** 10.1371/journal.pgph.0007264

**Published:** 2026-09-18

**Authors:** Karina Lalangui-Vivanco, Emmanuelle Quentin, Yekaterina Altuna-Roshkova, Juan Marcos Parise-Vasco, Jaen Cagua-Ordoñez, Claudia Reytor-González, Daniel Simancas-Racines, Ricardo Hidalgo

**Affiliations:** Centro de Investigación en Salud Pública y Epidemiología Clínica (CISPEC), Facultad Ciencias de la Salud Eugenio Espejo, Universidad UTE, Quito, Ecuador; PLOS: Public Library of Science, UNITED STATES OF AMERICA

## Abstract

Suicide is an important public health issue worldwide, especially among the young population. This study aimed to analyze the epidemiological characteristics and the spatial and temporal distribution of suicide at the provincial level in Ecuador between 2015 and 2023, using national mortality registries and spatiotemporal clustering methods. Crude rates were calculated by sex, age group, and province, and cluster analysis was applied using the K-means algorithm. A total of 10,297 suicides were recorded, with a clear predominance of males (78%), especially in the 20–29 age group, while among females the highest concentration occurred in the 10–19 age group in 2023. The most common method of suicide was hanging (73%). The highest rates were concentrated in provinces located in the Highlands (Sierra) and Amazon regions of the country. Five clusters of provinces were identified, with different levels and trends: (C1) provinces with very high but temporarily unstable suicide rates, which showed sharp peaks followed by sharp declines; (C2) provinces with persistently high rates during the study period; (C3) provinces with moderate and relatively stable rates over time; (C4) mainly coastal provinces with consistently low rates and minimal temporal variation; and (C5) the island region, characterized by a low rate with greater temporal dispersion. These findings highlight the need for territorially differentiated public policies, with emphasis on adolescent suicide prevention and improved access to mental health services.

## Background

Suicide is an important global public health problem in which people cause their own death through self-inflicted harm [1]. According to the Global Burden of Disease Study 2021, there were approximately 746,000 suicide deaths globally in 2021 [2].

Despite a 36% decline in the global suicide mortality rate between 2000 and 2019, the burden of this phenomenon remains disproportionate, as approximately 77% of suicides occur in low- and middle-income countries [3–5].

In demographic terms, suicide is one of the leading causes of death among adolescents worldwide, particularly in the 15–19 age group, while the highest mortality rates are consistently observed among adults aged 65 and older [1,6].

In the Americas, the trend runs counter to the global decline, with a 17% increase in suicide mortality over the past two decades [4]. In South America, an average of about 20,000 suicide deaths occur annually, with some variation between countries and subregions [4]. In the adolescent and young adult group (10–24 years), Central Latin America and Tropical Latin America showed a significant increase in suicide mortality between 1990 and 2021, with an Average Annual Percentage Change (AAPC) of 1.7 and 1.5, respectively [1,7].

In Ecuador, more than 1,000 deaths by suicide are recorded each year [8], and marked disparities have been identified in local epidemiology: between 2011 and 2021, the mortality rate among men was approximately four times higher than that among women, and suicide has become the leading cause of death among adolescent girls aged 13–20 [9,10].

In terms of methods, hanging is the predominant method in the country, accounting for more than 50–60% of cases [10]. In addition, notable differences between urban and rural areas have been documented: in particular, Ortiz-Prado et al. (2017) reported that urban communities had rates almost three times higher than rural communities, suggesting that urbanization, availability of resources, and access to health services may explain part of this disparity [10].

Although there are studies on suicide in Ecuador due to its importance as a public health problem [10–13], existing studies vary considerably in terms of scope, methodology, time coverage, and geographical focus [8,9,14].

While these studies provide valuable information and reflect methodological advances, our study takes a broader and complementary perspective: it analyzes relevant epidemiological variables by extending the time window to 2023 and applies spatiotemporal clustering methods at the province of residence level over a nine-year period. In this way, we seek to highlight the spatial and temporal trends in suicide at the provincial level from 2015 to 2023, providing updated evidence to support prevention and control strategies adapted to the territorial realities of Ecuador.

## Methods

### Study design and data source

This research was designed as a descriptive, observational epidemiological study with an ecological cluster approach.

Ecuador is a South American country administratively divided into 24 provinces (Fig 3A), with an estimated population of 16,938,986 inhabitants according to the 2022 Census. The provinces are grouped into four natural regions: the Coastal region (7 provinces), the Highlands or Sierra (10 provinces), the Amazon basin (6 provinces), and the Galápagos Islands (1 province). While smaller subdivisions exist (cantons and parishes), this study focused on provinces as the primary unit of analysis, as they represent a practical unit for public health planning.

The study period covered 2015–2023, spanning pre-pandemic, pandemic, and post-pandemic phases to capture recent temporal trends in suicide rates.

Data were obtained from the National General Registry of Deaths of the National Institute of Statistics and Census (INEC), which includes all deaths occurring in the country, excluding fetal deaths, which are reported in a separate database [15].

### Data preparation and statistical analysis

For ease of processing, the annual tables were merged into a single database, excluding records with a year of death outside the study period (extemporaneous records) and records of residents living abroad. Suicides were identified using codes X60 to X84 of the Tenth International Classification of Diseases (ICD-10), which correspond to intentional self-inflicted injuries.

Descriptive analysis was performed by sex and included the following variables: year of death, cause, age group, area of residence, region, day of death, level of education, civil status and ethnicity.

To improve the interpretability of the results, avoid categories with low frequency, and facilitate epidemiological comparisons, the variables for educational level, marital status, and ethnicity were recategorized into conceptually coherent groups. Differences between sex and the categorical variables were assessed using Pearson’s chi-square test, with a statistical significance level of p < 0.01.

Crude suicide mortality rates per 100,000 inhabitants were calculated for variables with available and comparable population denominators (sex, year, age group, province, region, and ethnicity). We noted the proportions of age and sex groups do not differ remarkably between the provinces of Ecuador, which justifies the calculation of the crude rate for this type of analysis, to avoid masking real rates [16].

### Spatiotemporal analysis

Crude suicide mortality rates were calculated for each year of the period 2015–2023 at the provincial level (Detailed results are provided in the S1 Table). In addition, a spatiotemporal clustering analysis was performed using the K-means++ algorithm implemented in the GeoDa software. This method was chosen for its suitability for continuous variables, its efficiency in initializing centroids and minimizing internal variance, and its reproducibility across multiple runs. This algorithm enabled the classification of the provinces into clusters with similar patterns over time, using the annual rates as input variables and keeping the default parameters of the software (50 reinitializations and a maximum of 1000 iterations) [17]. The elbow method was used to determine the optimal number of clusters. This consists of plotting the sum of squares within the clusters as a function of the number of clusters selected, identifying the optimal value at the point where the decrease in the sum of squares becomes less pronounced [18].

Data processing was performed using R software version 4.5.0. GeoDa 1.22 and Qgis 3.34.7 were used for the choropleth maps.

## Results

### Descriptive epidemiology of suicide mortality

A total of 10,297 suicide deaths were recorded between 2015 and 2023. Of which 8,027 (78%) occurred among men and 2,270 (22%) among women. The distribution of deaths by sex varied significantly across years (p = 0.003). The highest number of deaths was observed in 2016, whereas 2021 registered the lowest number (Table 1).

**Table 1:** Number and crude rates of suicides by sex and year in Ecuador, 2015-2023.

	Female		Male		Both sexes		p-value^2^
	N = 2,270*^1^*	Rate per 100,000	N = 8,027*^1^*	Rate per 100,000	N = 10,297*^1^*	Rate per 100,000	
**Year of death**							0.003
2015	272 (12%)	3.5	819 (10%)	10.8	1,091 (11%)	7.04	
2016	270 (12%)	3.4	965 (12%)	12.5	1,235 (12%)	7.87	
2017	244 (11%)	3.0	958 (12%)	12.3	1,202 (12%)	7.56	
2018	267 (12%)	3.3	954 (12%)	12.1	1,221 (12%)	7.58	
2019	249 (11%)	3.0	965 (12%)	12.1	1,214 (12%)	7.44	
2020	227 (10%)	2.7	866 (11%)	10.7	1,093 (11%)	6.61	
2021	182 (8%)	2.1	720 (9%)	8.8	902 (8.8%)	5.39	
2022	252 (11%)	2.9	908 (11%)	11	1,160 (11%)	6.85	
2023	307 (14%)	3.5	872 (11%)	10.5	1,179 (11%)	6.88	

^1^n = absolute frequency; ^2^Pearson´s Chi-squared test

**Fig 1:**
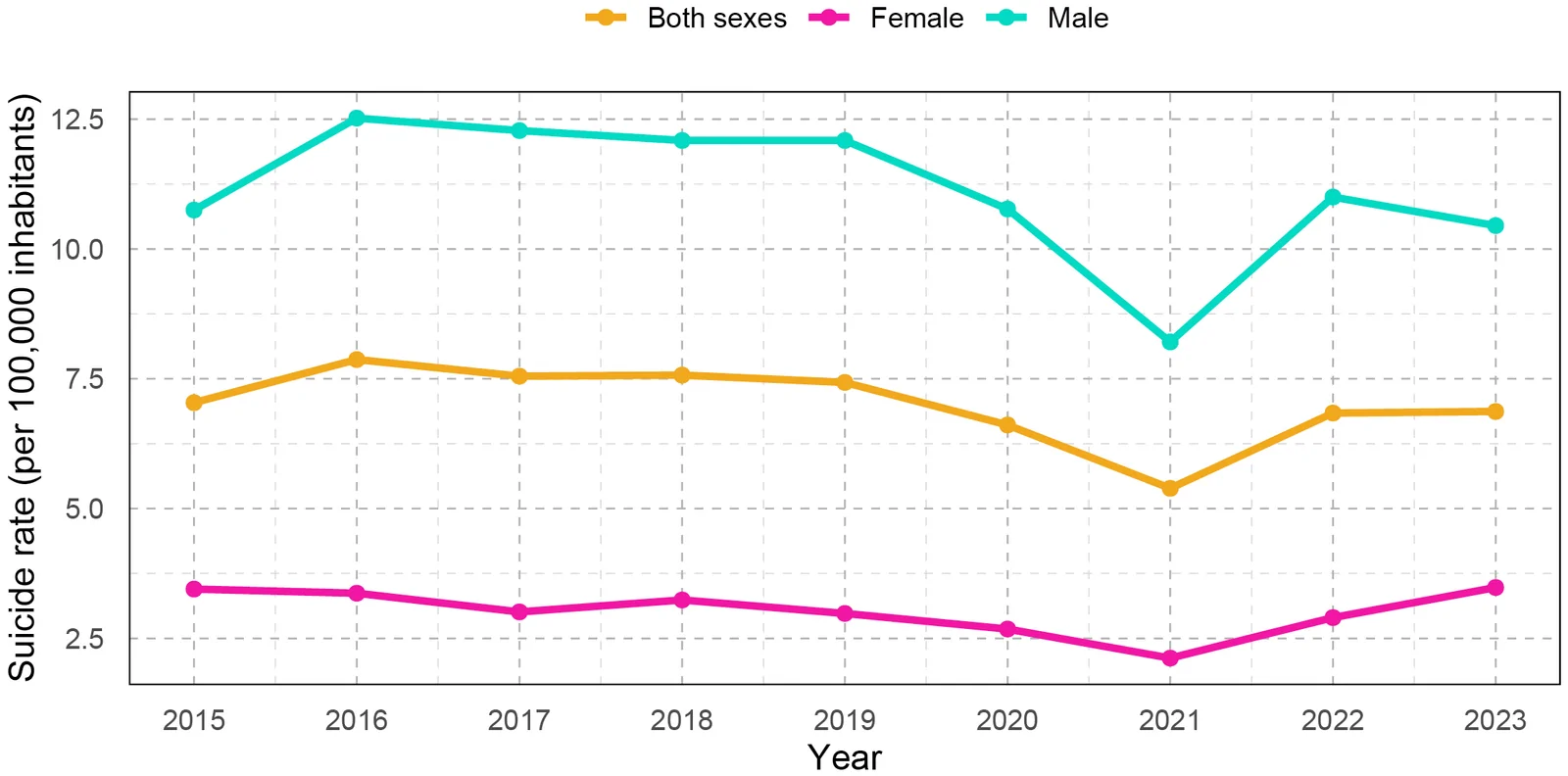
Crude suicide rate by sex in Ecuador, 2015-2023.

As for the crude suicide rates (Fig 1), over the years the rates in men were consistently higher than in women. In 2021, the lowest value was recorded for both sexes, followed by an increase in 2022. In 2023, the male rate decreased slightly (10.5 per 100,000), while the female rate increased (3.5 per 100,000).

The results showed that the majority of suicide deaths in Ecuador between 2015 and 2023 were concentrated among men (78%), while women accounted for 22% of cases. Table 2 showed the sociodemographic characteristics of suicide deaths in Ecuador. Statistically significant differences by sex were observed across age group, level of education, civil status, and ethnicity (p < 0.001 for all comparisons).

**Table 2:** Sociodemographic characteristics of suicide deaths in Ecuador, 2015-2023.

	Female	Male	Both sexes	p-value
	N = 2,270^*1*^ (22%)	N = 8,027^*1*^ (78%)	N = 10,297^*1*^	
**Age group**				<0.001
<10	4 (0.2%)	18 (0.2%)	22 (0.2%)	
10-19	815 (36%)	1,282 (16%)	2,097 (20%)	
20-29	568 (25%)	2,249 (28%)	2,817 (27%)	
30-39	345 (15%)	1,449 (18%)	1,794 (17%)	
40-49	182 (8%)	1,022 (13%)	1,204 (12%)	
50-59	142 (6.3%)	812 (10%)	954 (9.3%)	
60-69	115 (5.1%)	610 (7.6%)	725 (7%)	
70-79	64 (2.8%)	380 (4.7%)	444 (4.3%)	
80+	35 (1.6%)	204 (2.5%)	239 (3.2%)	
**Level of education**				<0.001
No education	89 (4.2%)	285 (3.8%)	374 (3.9%)	
Primary	1,001 (47%)	4,085 (54%)	5,086 (53%)	
Secondary	1,549 (9.1%)	5,032 (62.7%)	6,581 (63.9%)	
Tertiary	194 (7.5%)	568 (7.6%)	762 (7.9%)	
Unknown	136 (6%)	529 (6.6%)	665 (6.5%)	
**Civil status**				< 0.001
In a union	648 (29%)	2,600 (33%)	3,248 (32%)	
Not in a union	1,584 (71%)	5,255 (67%)	6,839 (68%)	
Unknown	38 (1.7%)	172 (2.1%)	210 (2%)	
**Ethnicity**				<0.001
Indigenous	209 (9.2%)	426 (5.3%)	635 (6.2%)	
Afro-descendant	41 (1.8%)	172 (2.1%)	213 (2.1%)	
Montubio	10 (0.4%)	134 (1.7%)	144 (1.4%)	
Mestizo	1,935 (85.2%)	6,975 (86.9%)	8,910 (86.5%)	
White	19 (0.8%)	58 (0.7%)	77 (0.8%)	
Other	4 (0.2%)	10 (0.1%)	14 (0.1%)	
Unknown	52 (2.3%)	252 (3.1%)	304 (2.9%)	

^1^n = absolute frequency; ^2^Pearson´s Chi-squared test

Most suicides occurred in the younger age groups. In men, most deaths were concentrated in the 20–29 age group (28%), while in women they occurred in the 10–19 age group (36%). The highest suicide rates (S2 Table) were observed in the 20–29 age group (92.8 per 100,000), the 70–79 age group (83.2 per 100,000), and those aged 80 and older (82.7 per 100,000). People with secondary education accounted for the highest proportion of cases (63.9%), and those not in a union represented 68% of the total. As for ethnicity, the mestizo group accounts for the highest proportion of suicides (86.5%), followed by the indigenous group (6.2%). Suicide rates by ethnicity were higher among mestizos (67.9 per 100,000) and Indigenous people (48.8 per 100,000) compared with people of African descent (26.2 per 100,000) and White people (20.5 per 100,000) (S2 Table).

Table 3 showed that the most common method for both sexes was hanging, accounting for 73% of all cases. Statistically significant differences by sex were observed in the distribution of suicide methods (p < 0.001). Among men, 74.8% of suicides were due to hanging, followed by poisoning (15%) and other methods (10.2%). In women, hanging was also the main method (67.5%), but poisoning also had a significantly higher proportion (26.8%), while other methods accounted for only 5.7%.

**Table 3:** Contextual characteristics of suicide deaths in Ecuador, 2015-2023.

	Female	Male	Both sexes	p-value^2^
	N = 2,270^*1*^ (22%)	N = 8,027^*1*^ (78%)	N = 10,297^*1*^	
**Cause**				<0.001
X70 (Hanging)	1,533 (67.5%)	6,007 (74.8%)	7,540 (73%)	
X60-X69 (Poisoning)	608 (26.8%)	1,205 (15%)	1,813 (17.6%)	
X71-X84 (Others)	129 (5.7%)	815 (10.15%)	944 (9.4%)	
**Area of residence**				<0.001
Urban	1,539 (68%)	5,795 (72%)	7,334 (71%)	
Rural	731 (32%)	2,232 (28%)	2,963 (29%)	
**Region**				<0.001
Amazonia	194 (8.5%)	546 (6.8%)	740 (7.2%)	
Cost	691 (30%)	3,354 (42%)	4,045 (39%)	
Insular	3 (0.1%)	13 (0.2%)	16 (0.2%)	
Sierra	1,382 (61%)	4,114 (51%)	5,496 (53%)	
**Day of death**				0.055
Monday	338 (15%)	1,180 (15%)	1,518 (15%)	
Tuesday	333 (15%)	1,080 (13%)	1,413 (14%)	
Wednesday	302 (13%)	1,014 (13%)	1,316 (13%)	
Thursday	313 (14%)	1,066 (13%)	1,379 (13%)	
Friday	317 (14%)	1,068 (13%)	1,385 (13%)	
Saturday	317 (14%)	1,144 (14%)	1,461 (14%)	
Sunday	350 (15%)	1,475 (18%)	1,825 (18%)	
**Month of death**				0.3
January	198 (8.7%)	733 (9.1%)	931 (9%)	
February	188 (8.3%)	677 (8.4%)	865 (8.4%)	
March	164 (7.2%)	694 (8.6%)	858 (8.3%)	
April	182 (8%)	654 (8.1%)	836 (8.1%)	
May	216 (9.5%)	686 (8.5%)	902 (8.8%)	
June	175 (7.7%)	669 (8.3%)	844 (8.2%)	
July	193 (8.5%)	683 (8.5%)	876 (8.5%)	
August	210 (9.3%)	659 (8.2%)	869 (8.4%)	
September	182 (8%)	648 (8.1%)	830 (8.1%)	
October	212 (9.3%)	652 (8.1%)	864 (8.4%)	
November	175 (7.7%)	653 (8.1%)	828 (8%)	
December	175 (7.7%)	619 (7.7%)	794 (7.7%)	

1 n = absolute frequency; ^2^Pearson´s Chi-squared test

Significant differences by sex were also identified for area of residence and region (p < 0.001 for both). Most suicides occurred in urban areas (71%), and the majority of deaths were concentrated in the Sierra region (53%). However, when crude rates were calculated by region (S2 Table), the highest suicide mortality rates were observed in the Amazon region (80.8 per 100,000), followed by the Sierra (77.8 per 100,000), whereas the Coastal region showed lower rates (44 per 100,000).

No statistically significant differences by sex were observed in the distribution of deaths by day of the week (p = 0.055) or month of death (p = 0.3). Although slightly higher proportions were observed during weekends, particularly on Sundays (18%), the overall distribution remained relatively stable throughout the week and across months. By sex, the highest percentages among men occurred in January (9.1%) and among women in May (9.5%).

A statistically significant association was observed between sex and province of residence (p < 0.001), indicating that the distribution of suicide deaths across provinces differed by sex. Men were overrepresented in Guayas, while women were overrepresented in Pichincha (Table 4).

**Table 4:** Distribution of suicide deaths by province of residence and sex in Ecuador, 2015–2023.

	Female	Male	Both sexes	p-value^2^
	N = 2,270^*1*^ (22%)	N = 8,027^*1*^ (78%)	N = 10,297^*1*^	
**Province of residence**				<0.001
Azuay	213 (9.4%)	686 (8.5%)	899 (8.7%)	
Bolívar	41 (1.8%)	118 (1.5%)	159 (1.5%)	
Cañar	52 (2.3%)	183 (2.3%)	235 (2.3%)	
Carchi	35 (1.5%)	97 (1.2%)	132 (1.3%)	
Chimborazo	121 (5.3%)	280 (3.5%)	401 (3.9%)	
Cotopaxi	105 (4.6%)	295 (3.7%)	400 (3.9%)	
El Oro	80 (3.5%)	406 (5.1%)	486 (4.7%)	
Esmeraldas	35 (1.5%)	178 (2.2%)	213 (2.1%)	
Galápagos	3 (0.1%)	13 (0.2%)	16 (0.2%)	
Guayas	305 (13%)	1,343 (17%)	1,648 (16%)	
Imbabura	87 (3.8%)	207 (2.6%)	294 (2.9%)	
Loja	69 (3%)	267 (3.3%)	336 (3.3%)	
Los Ríos	90 (4%)	456 (5.7%)	546 (5.3%)	
Manabí	95 (4.2%)	608 (7.6%)	703 (6.8%)	
Morona Santiago	35 (1.5%)	96 (1.2%)	131 (1.3%)	
Napo	23 (1%)	92 (1.1%)	115 (1.1%)	
Orellana	37 (1.6%)	112 (1.4%)	149 (1.4%)	
Pastaza	23 (1%)	49 (0.6%)	72 (0.7%)	
Pichincha	538 (24%)	1,625 (20%)	2,163 (21%)	
Santa Elena	14 (0.6%)	90 (1.1%)	104 (1%)	
Santo Domingo de los Tsáchilas	72 (3.2%)	273 (3.4%)	345 (3.4%)	
Sucumbíos	50 (2.2%)	139 (1.7%)	189 (1.8%)	
Tungurahua	121 (5.3%)	356 (4.4%)	477 (4.6%)	
Zamora Chinchipe	26 (1.1%)	58 (0.7%)	84 (0.8%)	

^1^n = absolute frequency; ^2^Pearson´s Chi-squared test

**Fig 2:**
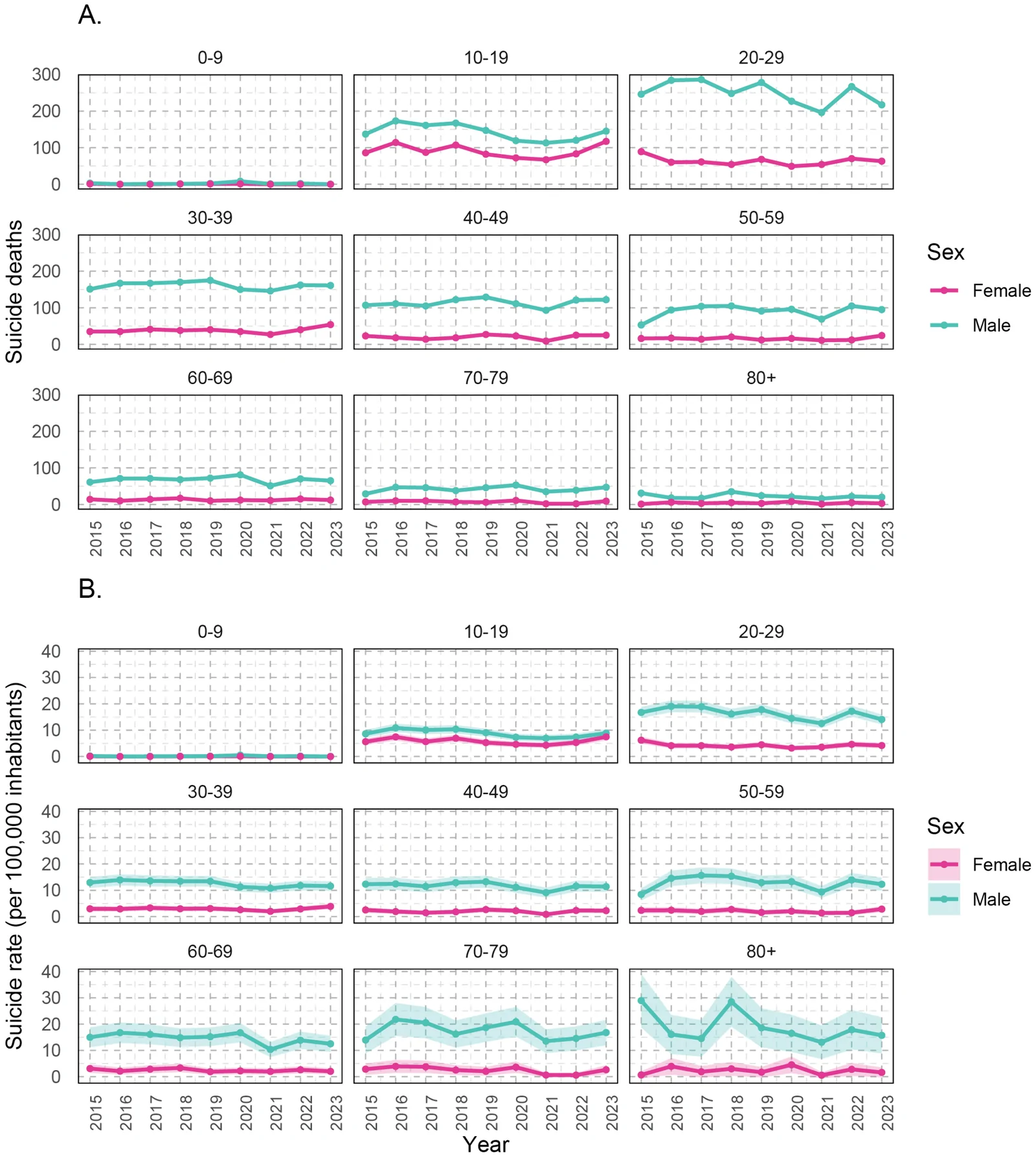
Annual distribution of suicide deaths and rates per 100,000 inhabitants, stratified by sex and age group in Ecuador, 2015-2023. Panel A shows the annual number of suicide deaths in Ecuador, disaggregated by sex and age group. Panel B presents suicide rates by age per 100,000 inhabitants, with shaded areas representing 95% confidence intervals.

The annual distribution of deaths and suicide rates by sex and age group between 2015 and 2023 (Fig 2) reveals consistent differences. In absolute terms (Fig 2A), men had a consistently higher number of deaths than women in all age groups, with a pronounced peak in the 20–29 age group. In contrast, among women, the 10–19 age group stands out as the most affected.

When analyzing age-specific rates (Fig 2B), it can be seen that men also recorded higher values throughout the period, especially among adults and older adults, where the relative risk is higher than among young people. However, in absolute terms, the number of deaths in these adult groups was lower than in the younger groups.

An important point to note is that in both sexes, an upward trend was identified from 2021 onwards in the 10–19 age group.

### Spatiotemporal distribution of suicide

**Fig 3:**
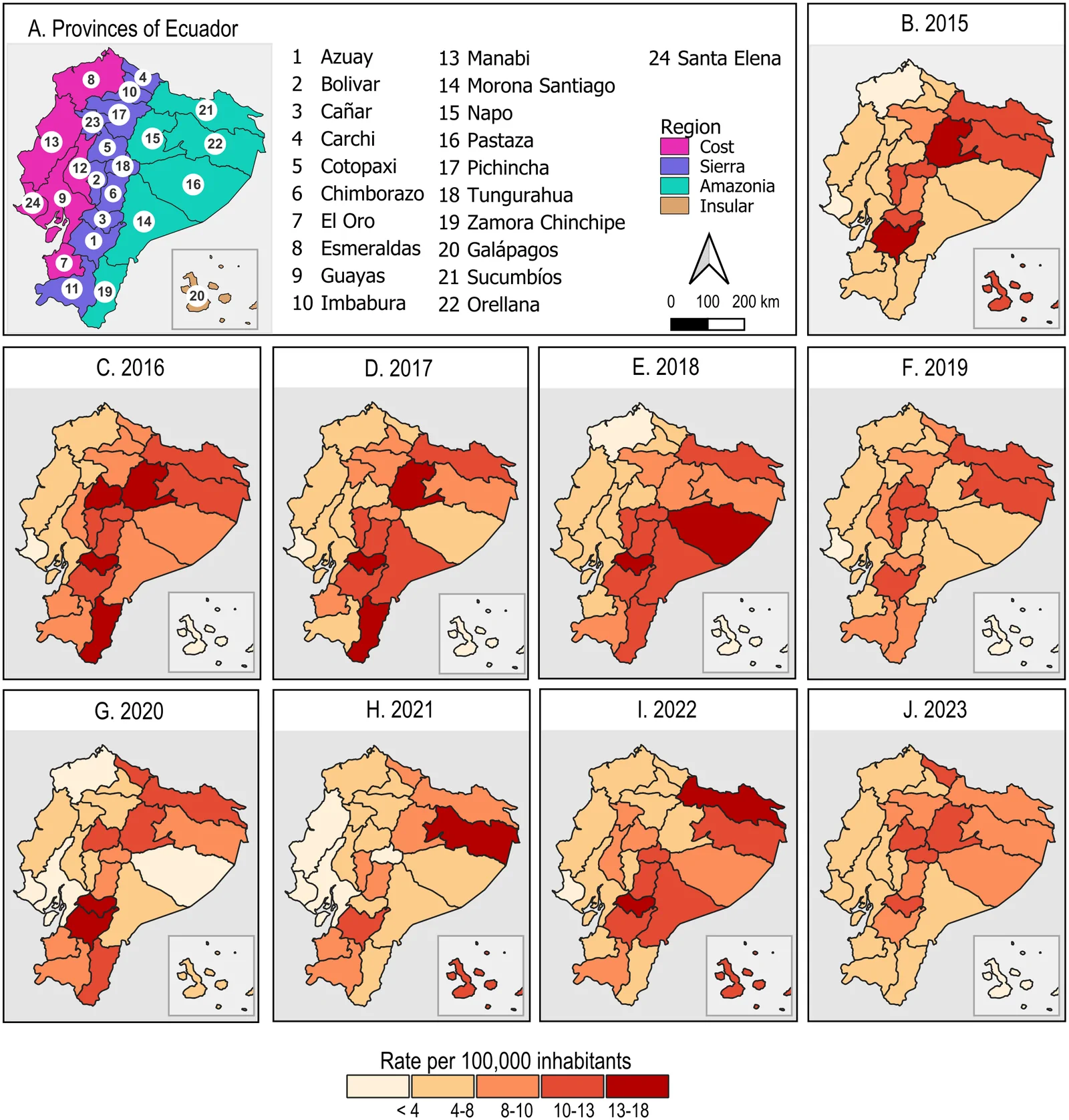
Geographical location of the 24 provinces of Ecuador and crude suicide death rate by province of residence per year, 2015 to 2023. Location of Ecuador’s 24 provinces (A). Annual choropleth maps showing provincial variation in suicide rates (per 100,000 inhabitants) over the period 2015–2023 (B). Rates were grouped into intervals to facilitate comparison between years. Base map: Natural Earth Admin 1 – States, Provinces (public domain). Source: https://www.naturalearthdata.com/downloads/10m-cultural-vectors/10m-admin-1-states-provinces/.

The annual choropleth maps (Fig 3B-J) reveal spatial and temporal variability in suicide mortality rates across Ecuador between 2015 and 2023. Throughout the study period, provinces located in the highlands consistently showed higher rate categories compared to most coastal provinces, which remained predominantly within the lower rate ranges.

The Amazon region showed marked year-over-year fluctuations, with certain provinces reaching the highest rate categories in 2015, 2016, 2018, and 2022.

The spatial concentration of provinces with high rates is particularly evident from 2016 to 2018, when several provinces simultaneously reached the highest rate ranges (13–18 per 100,000). In contrast, in 2021 and 2023, a general reduction in rates is observed in most provinces, with fewer areas in the highest categories. Although some spatial patterns appear persistent, especially in the Central Highlands, the Amazon region shows greater temporal instability throughout the period.

**Fig 4:**
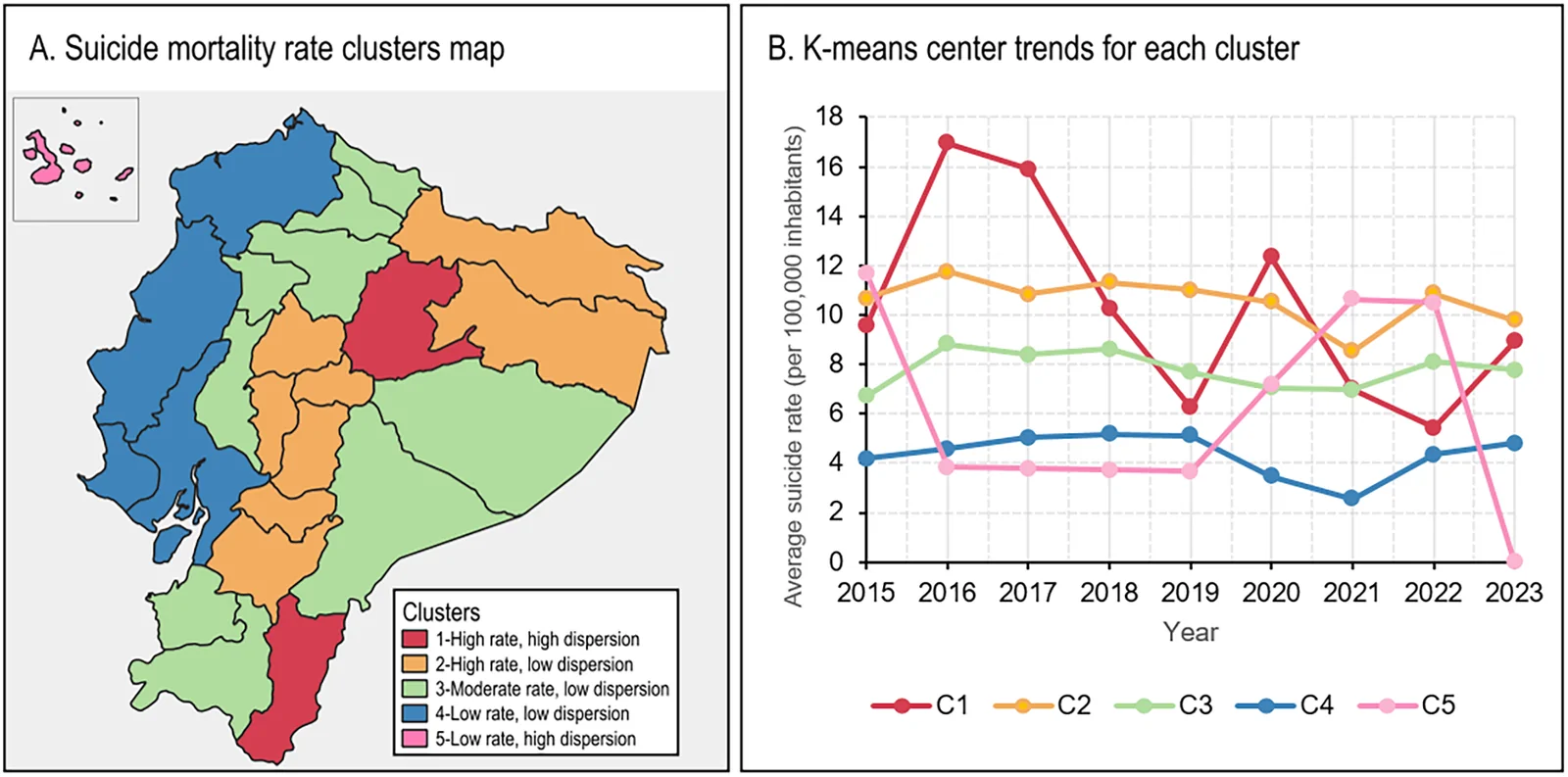
Suicide mortality rate clusters. The map shows the provinces with similar patterns in their suicide mortality rates over the period 2015–2023. The graph presents the average suicide rates in the grouped provinces for each year. Base map: Natural Earth Admin 1 – States, Provinces (public domain). Source: https://www.naturalearthdata.com/downloads/10m-cultural-vectors/10m-admin-1-states-provinces/.

Cluster analysis performed on the crude provincial suicide rates over this period identified five clusters with distinct characteristics. Cluster 1 included provinces such as Napo and Zamora Chinchipe, which had high suicide rates but with a marked dispersion over time, reaching a significant peak in 2016 (17.1 and 16.9, respectively), followed by a notable decline in subsequent years. Cluster 2 included provinces such as Cotopaxi, Bolivar, Tungurahua, Chimborazo, Cañar and Azuay, characterized by consistently high suicide rates, which peaked in 2016 and remained high for most of the period, fluctuating between 8.6 and 11.7. Cluster 3 grouped provinces with moderate suicide rates and little variation over time (between 6.7 and 8.8). Cluster 4 was found mainly in the coastal provinces, where suicide rates were low and with little dispersion, ranging from 2.5 to 5.2. Finally, cluster 5 included the Insular region, where rates were low with greater dispersion (Table 5, Fig 4).

**Table 5:** Crude rates of suicide deaths and spatiotemporal groupings.

Province	Rate per 100,000 inhabitants									Cluster code
	2015	2016	2017	2018	2019	2020	2021	2022	2023	
Napo	13.1	17.1	14.2	9	4	12.6	8.5	4.6	10.6	C1
Zamora Chinchipe	6.1	16.9	17.6	11.5	8.5	12.1	5.5	6.3	7.2	C1
Azuay	13.4	11.9	12.8	12.3	12.5	15.8	12.2	12.6	9.7	C2
Bolívar	12.6	12.5	11.4	11.9	12.8	5.1	9.1	5	7.5	C2
Cañar	10.6	13.3	15	14.1	9.3	14.5	4.8	13.6	12.3	C2
Chimborazo	8.6	10.5	10.7	10.5	7.9	8.7	8.5	11.9	8.3	C2
Cotopaxi	7.4	13.4	6.3	7.8	13	12.2	8	8.3	11.5	C2
Orellana	11.7	10.1	9.9	9	10.6	8.6	15.7	11	9.3	C2
Sucumbíos	10.3	10.2	10.6	12.1	10.9	10.3	9.1	13.1	9.6	C2
Tungurahua	10.8	12	9.9	12.9	11.3	9	0.9	11.5	10.1	C2
Carchi	6.6	9.5	7.7	7.7	8.8	12.8	8.7	7.5	11.6	C3
El Oro	7.3	8.7	8.6	7.4	7.4	8.3	8.4	7.4	7.4	C3
Imbabura	7.7	9.9	9.3	6	6.8	4.1	6.7	6.2	7.7	C3
Loja	6.7	9.2	6.6	8.2	8.2	8.8	8.7	9.7	5.6	C3
Los Ríos	6.5	8.5	5.8	7.9	8.2	6.3	6.6	8	7	C3
Morona Santiago	6.6	10	12.7	10.7	7.7	7	4.2	10.9	4.2	C3
Pastaza	4.2	9.2	8	13.7	5.7	2.8	5.5	8	9.8	C3
Pichincha	8.1	8.7	8.6	8.8	9.2	7.2	7.4	6.4	8.5	C3
Santo Domingo de los Tsáchilas	7	5.8	8.5	7.1	7	6.1	6.6	8.9	8.3	C3
Esmeraldas	3.5	5.2	5.1	3.6	6.3	2.8	4.2	5.1	5.6	C4
Guayas	5.2	4.9	5	4.4	4.3	3.7	2.1	4.3	4.8	C4
Manabí	4.9	6	6.4	7.7	6.6	4.7	1.8	5.2	4.5	C4
Santa Elena	3.2	2.3	3.7	5	3.3	2.7	2.1	2.9	4.4	C4
Galápagos	11.7	3.8	3.8	3.7	3.7	7.2	10.7	10.5		C5

## Discussion

This study provided a comprehensive characterization of the epidemiological and spatiotemporal patterns of suicide in Ecuador between 2015 and 2023, showing significant differences by sex, age and geographic distribution.

A higher incidence of suicide was observed in young men aged 20–29 years and in adolescent women aged 10–19 years, with a predominance of the hanging method and a higher concentration of cases in the Sierra provinces. The grouping analysis using the K-means algorithm allowed the delimitation of five provincial clusters with differentiated temporal and spatial behaviors, reflecting a heterogeneous territorial configuration in the occurrence of suicide in the country.

These findings are partially consistent with the international literature, where hanging has also been reported as the most common method in Latin American countries, especially in rural areas of Brazil and Mexico [19,20]. In fact, in the Region of the Americas, self-harm by hanging, strangulation, or suffocation increased from 34.1% in 2000 to 47.3% in 2017 as a proportion of total suicides [4].

Significant differences were also observed in the age distribution of cases. While the highest absolute number of suicides is concentrated among young people, specifically among women aged 10–19 (36% of all cases) and men aged 20–29 (28% of all cases), suicide mortality rates reach their highest levels among older adults (Fig 2).

This distinction between absolute burden and relative risk is fundamental: older adults represent the group with the highest individual probability of dying by suicide, but the social and health impact falls mainly on young people, who account for the highest proportion of deaths and years of life lost (YLLs) [20]. In Mexico, for example, although rates were higher among older adults, the burden of disease from suicide increased mainly among young people, with more than 77% of YLLs concentrated between the ages of 15 and 45 [20].

Similarly, at the global level, although suicide rates among adolescents and young adults (aged 10–24) declined from 1990 to 2021, regions such as Central Latin America (AAPC: 1.7%) and Tropical Latin America (AAPC: 1.5%) showed a significant increase in suicide mortality in this age group [1].

The results also highlight the influence of socioeconomic determinants such as educational level, marital status, and ethnicity on suicide. More than 80% of deaths were concentrated among people with primary and secondary education, which aligns with studies that consistently show that lower educational attainment is associated with a higher risk of suicide [21], due to the relationship between precarious working conditions or unemployment [5]. However, it is important to emphasize that in the study by Ortiz-Prado et al. (2024), a more complex trend was observed: individuals with higher educational levels (technical and university education) showed significantly higher suicide rates at high altitudes, suggesting that factors such as altitude and other socioeconomic and cultural determinants need to be explored in greater depth [9].

In terms of marital status, people without a partner had the highest suicide rate (71%). This data reinforces international evidence suggesting that being married or cohabiting with a partner acts as a protective factor, while being single, divorced, or widowed increases the risk of suicide due to the absence of emotional support and close family networks [22].

With regard to ethnicity, the study shows that the majority of suicide deaths occurred among the mestizo population (86.5%), which partly reflects the national demographic composition. However, crude rates indicate that both the mestizo and indigenous populations experience high suicide mortality (67.9 and 48.8, respectively). Although the proportion of deaths among indigenous people (6.2%) is lower in absolute terms, their suicide rate remains substantial and consistent with evidence from other Latin American contexts. Previous research has documented higher suicide rates among indigenous communities, associated with territorial marginalization, discrimination, cultural loss, and barriers to accessing health services [23]. In Ecuador, a recent analysis of suicide prevalence in the Amazonian provinces of Napo and Orellana [8] confirms that “being of indigenous ethnicity is relevant to the tendency to commit suicide.”

Temporal variations in suicide mortality were also observed, with the highest rates recorded in 2016 (7.9 per 100,000) and the lowest in 2021 (5.4 per 100,000). The peak observed in 2016 is consistent with the 17% increase that the Region of the Americas experienced between 2000 and 2019 [4].

In contrast, the sharp decline in 2021 coincides with the COVID-19 pandemic, a period during which specific studies from Ecuador reported that suicide rates were no higher than expected; rather, it has been suggested that lockdown measures may have acted as a protective factor [24]. However, this decline was followed by an increase in subsequent years, suggesting delayed psychosocial consequences of the pandemic, such as economic hardship, prolonged social isolation, and reduced access to mental health services. Similar increases have been reported internationally in the wake of the pandemic, supporting the hypothesis that COVID-19 has delayed effects on suicidal behavior., particularly among adolescents and women [25–28].

The spatiotemporal approach adopted allowed us to visualize the geographic distribution of suicide risk, revealing a persistent concentration in Andean and Amazonian provinces. This pattern is congruent with previous research that has documented how conditions of territorial marginality, multidimensional poverty, limited coverage of mental health services, and less institutional connectivity influence the appearance of clusters of high suicide frequency in rural and dispersed contexts [29,30].

Environmental factors such as altitude have also been explored in relation to suicide. In Ecuador, Ortiz-Prado et al. (2024) [9] reported age-standardized suicide rates that were almost double in cantons at moderate to very high altitudes compared to low-lying areas (4.3-4.4 versus 2.5 per 100,000 inhabitants). These findings are consistent with other international literature [31] and highlight the possible influence of geographical and environmental determinants.

In this sense, cluster-level studies provide an initial basis for identifying territorial patterns of risk and generating relevant hypotheses. Although a biological explanation related to oxygen levels at higher elevations has been proposed in relation to depression, elevation may act as a confounding factor rather than a causal one. This is further suggested by our findings, which show high suicide rates in the Amazon region—despite its elevation being similar to that of the coastal areas.

Additional research at the individual level with higher geospatial resolution is required. The lower burden observed in the coastal provinces may be related to protective factors that have not yet been studied in depth, such as community networks, greater service provision or differentiated social dynamics.

From a public health perspective, these results support the need for policies that are differentiated by age group, sex and territory, identifying the central Sierra and Amazonian provinces should be priority areas for intervention.

The risk of suicide is strongly influenced by sociocultural and structural determinants [9]. Among the most relevant social determinants are socioeconomic inequality, educational level, rurality, ethnicity, age-related discrimination, and social isolation [9]. For example, in Ecuador, higher rates have been observed in high-altitude rural areas and among indigenous, mestizo, and montubio populations [9].

In this regard, population policies must adopt an approach that encompasses the entire government and society, integrating universal, selective, and targeted interventions [5]. These strategies should strengthen access to mental health services in marginalized regions, incorporate culturally adapted measures differentiated by sex and age, and include school-based actions such as early screening, programs to promote emotional well-being, and the strengthening of community mental health services [32].

Recent evidence highlights the effectiveness of multisectoral and multilevel approaches that integrate universal, selective and indicated interventions, especially when implemented in school and community settings and with the participation of local stakeholders [33].

With regard to scientific research, this study identifies strategic lines for its deepening. It is a priority to move towards more complex methodological designs, including mixed and longitudinal approaches, capable of capturing psychosocial, economic and cultural dimensions that modulate suicidal behavior in specific contexts [34].

There is also a need to generate evidence on groups that have been historically invisible in population studies, such as LGBTIQ+ youth, indigenous peoples, migrants, and socially excluded youth [35]. The integration of contextual and qualitative variables will allow for a more nuanced understanding of suicide and facilitate the design of culturally relevant and territorially sensitive interventions.

In addition, the results of this study underscore the value of the ecological approach and spatiotemporal analysis as effective tools for identifying high-risk territorial clusters and generating useful evidence for programmatic planning. As discussed in recent evaluation frameworks, these approaches need to be complemented by population-based longitudinal studies and multilevel methods to explore interactions between individual and contextual characteristics [36].

It is also recommended to use mixed designs with qualitative methods to explore perceptions, barriers and facilitators in high burden areas, as well as the acceptability of the interventions implemented. Evaluation of public prevention policies requires a broader methodological framework, including intermediate indicators, analysis of implementation and monitoring of outcomes in specific populations, especially young people and historically excluded groups [35]. Potentially useful methodological tools, depending on the type of intervention, territorial level and data availability, include hierarchical Bayesian models, smoothed relative risk analysis and interrupted time series, which can help to better estimate programmatic effects and control for contextual variations [5].

Despite these methodological considerations, this study offers several important contributions. It provides national coverage over a nine-year observation period, which allowed for the identification of temporal trends and spatiotemporal clustering with sufficient robustness. The analysis stratified by sex and age groups provides relevant information on the largest and most at-risk population subgroups. The use of spatiotemporal cluster analysis (K-means++) represents a novel application in the study of suicide in Ecuador, allowing the identification of territorial patterns that are very useful for guiding prevention policies. It is also worth noting that the provincial scale of the analysis allows for the detection of structural trends, regional disparities, and actionable patterns that would not be visible or addressable at the scale of smaller administrative units. This balances analytical reliability with practical relevance for planning and prevention programs. It also makes it easier for public health authorities to identify priority areas and implement strategies that reach the entire province. This offers a broader range of feasible mental health-related interventions.

### Limitations

This study has several limitations that should be considered when interpreting the findings. First, the ecological design does not allow for causal inferences at the individual level. Second, although official death records are the most comprehensive source of mortality data in Ecuador, they may be subject to underreporting, misclassification of causes, or coding errors, especially in rural or stigmatized areas where suicides may be concealed or incorrectly recorded. Third, from a public health perspective, this study conducts its spatial analysis at the provincial level, using provinces as the unit of aggregation based on the deceased’s usual place of residence. While this level is relevant for decision-making and resource allocation, it may mask important within-province variations. Future studies could therefore consider finer levels of geographic aggregation, particularly in high-burden provinces, to better capture local patterns. Finally, the absence of information on psychosocial and clinical determinants limits a comprehensive understanding of the phenomenon, which is a priority for future research with complementary designs.

## Conclusions

The descriptive and spatiotemporal analysis of suicide in Ecuador during the period 2015–2023 revealed patterns that require priority attention in public health. The 10,297 deaths recorded, with a marked male predominance in young populations, especially men aged 20–29 years and women aged 10–19 years, demonstrate the magnitude of this problem. The notable increase in the number of cases among adolescents (10–19 years) in 2023 indicates an emerging trend that requires immediate intervention.

Cluster analysis identified five distinct spatial patterns, highlighting high-risk areas in the central Sierra and in the Amazon, while the coastal region has consistently lower rates. This geographic variability underscores the need for differentiated strategies according to the regional context.

In light of these findings, there is a clear need to implement preventive strategies that address different dimensions of the problem.

These strategies include: school programs focused on early detection and prevention of adolescent suicide, strengthening mental health services, and restricting access to lethal means. Future research should incorporate mixed methods to better understand the protective factors present in regions with lower rates, as well as to deepen the analysis of psychosocial and economic determinants specific to each regional context.

## Supporting information

S1 Table Crude suicide death rates by province in Ecuador, 2015–2023. (XLSX)

S2 Table Suicide death rates by sex, year, age group, region, ethnicity, and province of residence in Ecuador, 2015–2023. (XLSX)
